# The development of a rapid SYBR one step real-time RT-PCR for detection of porcine reproductive and respiratory syndrome virus

**DOI:** 10.1186/1743-422X-7-90

**Published:** 2010-05-10

**Authors:** Hong Tian, JingYan Wu, YouJun Shang, Yan Cheng, XiangTao Liu

**Affiliations:** 1 Key Laboratory of Animal Virology of Ministry of Agriculture, State Key Laboratory of Veterinary Etiologic Biology, Lanzhou Veterinary Research Institute, Chinese Academy of Agricultural Sciences, Lanzhou, Gansu730046, China

## Abstract

**Background:**

Prompt detection of PRRSV in the field samples is important for effective PRRS control, thereby reducing the potentially serious economic damage which can result from an outbreak. In this study, a rapid SYBR-based, one step real-time RT-PCR quantitative reverse transcription PCR (qRT-PCR) has been developed for the detection of porcine reproductive and respiratory syndrome virus (PRRSV). Primers were designed based on the sequence of highly conservative region of PRRSV N gene.

**Results:**

The sensitivity of the real-time qRT-PCR assay was achieved through PRRSV ch-1a RNA for the generation of a standard curve. The detection limit of the assay was found to be 9.6 RNA copies per reaction mixture. This assay had excellent intra- and inter-assay reproducibility as in total 65 field samples were screened for the presence of PRRSV by conventional RT-PCR in parallel with qRT-PCR, and the detection rate increased from 60.0% to 76.9%. Moreover, the specificity result indicated that this assay could reliably differentiate PRRSV from the other swine viral diseases, such as classical swine fever virus (CSFV), swine vesicular disease virus (SVDV) and vesicular exanthema of swine virus (VESV).

**Conclusion:**

The real-time qRT-PCR assay described in this report allows the rapid, specific and sensitive laboratory detection of PRRSV in field samples.

## Background

Porcine reproductive and respiratory syndrome virus (PRRSV) is a member of the family *Arteriviridae *[1], and it was characterized by respiratory disease in young pigs and severe reproductive failure in sows, including abortion, stillbirths and weak piglets [2]. PRRS has caused immense economic losses in the pig industry and is considered to be one of the most important infectious diseases of pigs in the world [3]. PRRSV has been recognized as one of the most important pathogens of pigs throughout the world [4]. This virus was first confirmed in China in 1996, since then, the virus has spread widely in China [5,6].

Prompt detection of PRRSV in the field samples is important for effective PRRS control, thereby reducing the potentially serious economic damage which can result from an outbreak. Therefore, rapid, specific and sensitive assays are required for diagnosis of PRRSV in pigs. Isolation of virus in cell cultures is technically difficult and time-consuming and thus is not suitable for routine diagnostic assay. Since 1995, several reverse transcription-PCR (RT-PCR) methods have been developed for the rapid and specific detection of PRRSV in pigs; however, these conventional RT-PCR assays are labor intensive, as they require gel analysis for the PCR products, and they are not suitable for high throughput testing. In contrast, real-time RT-PCR, which completes amplification and analysis in a closed system, has many advantages over conventional RT-PCR methods: lower chance of contamination, allows quantitative measurement of RNA, more rapid to perform and higher sensitivity.

The aims of this study were to develop a rapid and sensitive method to detect a wide range of field samples of PRRSV in pigs within a short period of time. In this study, we used a one step SYBR green real-time RT-PCR method to detect and quantify PRRSV from field samples. The results indicated that this method provide a new avenue to the rapid detection of PRRSV in one reaction.

## Results

### Optimization of one step SYBR green real-time RT-PCR

The annealing temperature and the primer concentration were optimized (Tables 1 and 2) using the RNA extracted from ch-1a (viral RNA concentration 9.6 × 10^5 ^copy numbers per reaction mixture) as a positive control. The optimal annealing temperature was 56°C, and optimal primer concentrations were 0.4 μM. The results were analyzed using MxPro™ QPCR Software and Tm values were taken to verify the specificities of the PCR products.

**Table 1:** Annealing temperature of Nf/Nr oligonucleotide pair optimization by means of Ct values

Annealing temperature	Cycle threshold (Ct)
53	23.12
55	22.90
56	21.26
57	23.78
58	25.62

**Table 2:** Determination of optimized primers concentration for the PRRSV one step real-time RT-PCR

primers concentration (μM)	Cycle threshold (Ct)
0.1	> 40
0.2	37.11
0.3	29.05
0.4	25.60
0.5	25.21
0.6	24.90

### Reproducibility of one step SYBR green real-time RT-PCR

**Figure 1:**
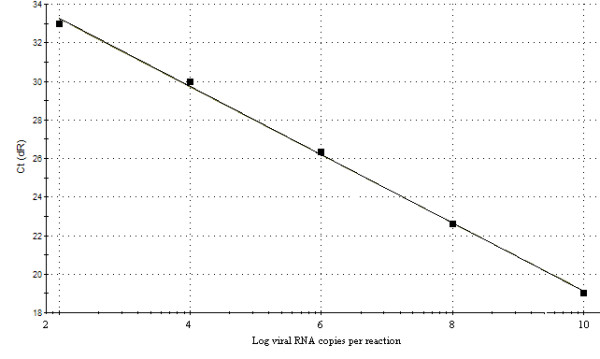
**Standard ch-1a curve of the PRRSV real-time qRT-PCR assay**. A 10-fold serial dilutions ranging from 9.6 to 9.6 × 10^5 ^copies of PRRSV RNA were tested in the real-time qRT-PCR. The standard formula is y = - 3.528x + 15.60 and the correlation co-efficient is 0.999.

In order to evaluate the reproducibility, a dilution end-point standard curve was made and was repeated for three times. Ct values were measured in triplicate and were plotted against the amount of infectious units (Fig. 1). The inter-assay calibration curves in Fig. 1 indicate that a linear detection range between 9.6 to 9.6 × 10^5 ^copy numbers per reaction mixture. The standard formula is y = -3.528x + 15.60 and the correlation co-efficient is 0.999.

### Analytical specificity and sensitivity of one step SYBR green real-time RT-PCR

**Figure 2:**
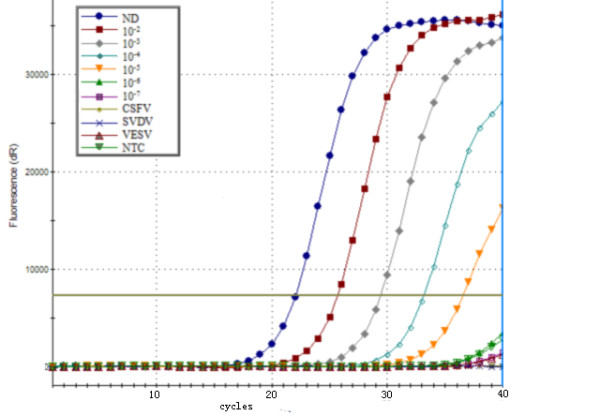
**The specificity and sensitivity of one step SYBR green real-time RT-PCR**. Plot of the amplification of a 10-fold serial dilution of ch-1a RNA to calculate the detection limit and sensitivity of real-time RT-PCR by analyzing the fluorescence curve of the 228 bp DNA amplification product. NTC is the negative control; ND is the non-diluted sample (9.6 × 10^5^); CSFV is classical swine fever virus; SVDV is swine vesicular disease virus; VESV is vesicular exanthema of swine virus; ch-1a dilutions are 10^-2 ^to 10^-7 ^with copies from 9.6 × 10^5 ^down to 9.6 per reaction mixture.

**Figure 3:**
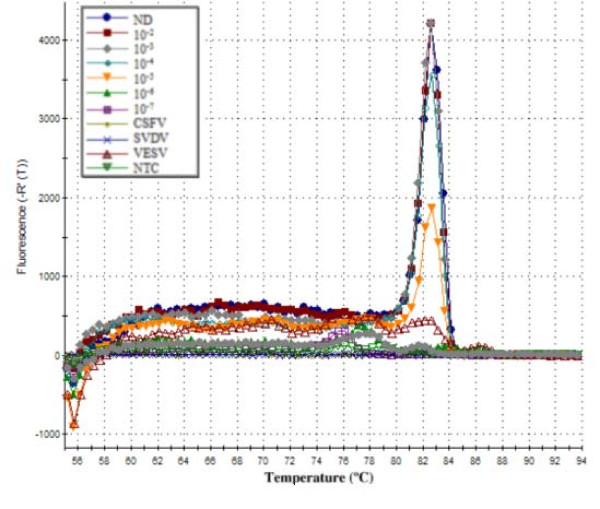
**Dissociation plot of the amplification products of ch-1a RNA**. Negative control including NTC, CSFV, SVDV AND VESV, melting peaks of ch-1a ten-fold serial dilutions and negative control, the positive samples showed an identical melting curve profile.

PRRSV, CSFV, SVDV and VESV strains from widely different geographical regions were selected for amplification in the qRT-PCR assay. We found that specific signal was found in PRRSV but not in CSFV, SVDV and VESV. To determine the sensitivity of the one step SYBR green real-time RT-PCR method, ch-1a RNA were diluted 10-fold serially from undiluted to10^-6^. The diluted ch-1a was still positive at 10^-5 ^dilution (Ct = 38.65), the sensitivity of this method was 9.6 copy numbers per reaction mixture. No primer-dimers or non-specific product were found in negative control (Fig. 2 and Fig. 3).

### Comparison of real-time qRT-PCR and conventional RT-PCR

**Figure 4:**
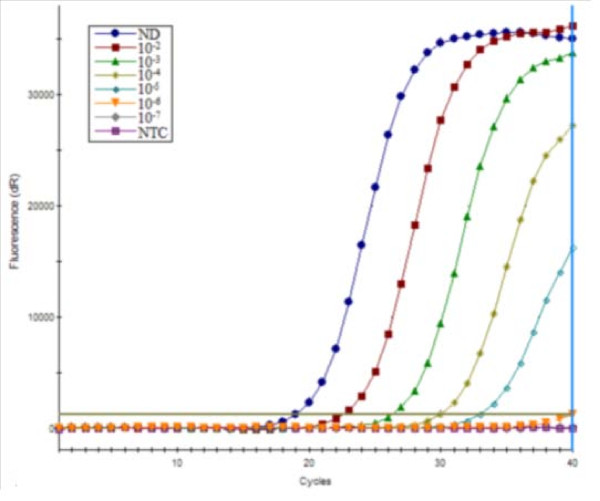
**The sensitivity of real-time qRT-PCR for detection of the PRRSV**. A 10-fold dilution series of total RNA extracted from a field sample ranging from 10^-1 ^to 10^-7 ^dilutions were tested in parallel in the qRT-PCR assay and in the conventional RT-PCR. The detection limit for the real-time qRT-PCR assay was a 10^-5 ^dilution of sample RNA.

**Figure 5:**
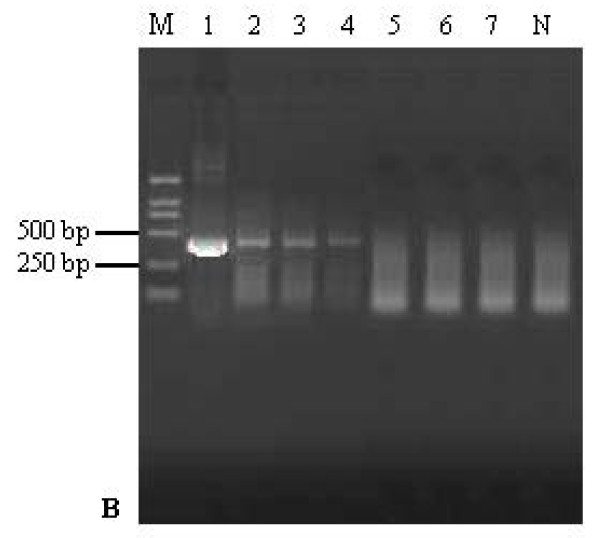
**The sensitivity of conventional RT-PCR for detection of the PRRSV**. The detection limit for the conventional RT-PCR assay was a 10^-4 ^dilution of sample RNA. Lanes 1--7: 10-fold dilution series of sample total RNA ranging from 10^-1 ^to 10^-7 ^dilutions; lane N: negative control having no template; lane M: DL2000 DNA ladder Maker (TAKARA).

Next, the detection limit of the real-time qRT-PCR assay and the conventional RT-PCR assay were compared. The real-time qRT-PCR assay was able to detect a 10^-5 ^diluted sample, with a corresponding Ct value of 38.65 ± 0.49 (Fig. 4). In parallel, the analytical sensitivity of the conventional RT-PCR was found to be a 10^-4 ^dilution (Fig. 5). These results indicate that the sensitivity of the qRT-PCR assay was observed to increase one log unit than that of the conventional RT-PCR assay.

### Detection in field specimens

To assess the use of the qRT-PCR for the detection of viral RNA in the field, 65 samples were collected as described previously. 39 of these 65 samples were found to be positive by conventional RT-PCR, and viral RNA loads were found to be in the range of 10^2 ^to 10^4 ^copies per reaction mixture (2 μl total RNA). Further, qRT-PCR was performed to all these 65 samples. Surprisingly, eleven additional samples were identified positive by this assay (Table 3). These additional samples were confirmed to be positive by sequence analysis. These data further confirmed that our method is more sensitive than the traditional method in the detection of field specimens.

**Table 3:** Diagnostic field samples tested positive by real-time qRT-PCR or conventional RT-PCR

Tissue samples	Number	Conventional RT-PCR positive	Real-time qRT-PCR positive
Lung	40	36	39
Lymphoid tissues	10	2	5
Spleen	7	1	3
Liver	8	0	3
Total	65	39	50

## Discussion

Real-time RT-PCR has several advantages over conventional RT-PCR. Firstly, it is more rapid and sensitive. Secondly, it is performed in a closed one-tube system and avoids potential cross contamination during sample preparation for post-PCR analysis. Real-time RT-PCR assays have been widely utilized for early diagnosis of many other animal viral diseases [7,8]. In this study a one-step real-time RT-PCR assay was developed and evaluated for detection of PRRSV in field samples. The assay described in this report generates complete result within 2 h and can be used as a rapid diagnostic tool.

To improve the specificity and sensitivity of the method described it was necessary to optimize the conditions of primers and annealing temperature. With these parameters, the detection of the ch-1a could be up to a 10^-5 ^dilution. This method does not require post-PCR manipulation, because the melting curve data allow us to verifying the amplification products diminishing the potential contamination risk. No primer-dimers were observed in the amplification products when analyzed by melting curve. Under the conditions mentioned in this paper, the sensitivity of this method was 9.6 copies per reaction mixture (Fig. 2). By comparing with conventional RT-PCR, the analytical sensitivity was found to be a 10^-4 ^dilution. It is obvious that our method can increase the sensitivity one log unit than that of the conventional RT-PCR assay.

Considering the prevalence and economic impact of PRRSV, a simple, cost effective, sensitive and rapid diagnostic technique is very important. The one step SYBR green real-time RT-PCR assay described in this study has all these attributes. This technique has tremendous applications in routine diagnostics in common laboratories.

## Conclusion

The one-step real-time RT-PCR assay described in this report appears to be a simple, sensitive, specific and rapid method for detection and quantitation of PRRSV in field samples

## Materials and methods

### Virus strain and field samples

CH-1a (GenBank access number: AY032626) virus strain, CSFV, SVDV and VESV were preserved at virology department of Lanzhou Veterinary Research Institute, Gansu of P.R.China. 65 field samples were collected from PRRSV-suspected animals during 2008 in China. Samples were placed at -70°C for further use.

### Viral RNA extraction

The viral RNA of all field samples was extracted by using QIAamp Viral RNA Mini Kit (Qiagen). In brief, after lysis of the specimens, the mixture was applied to a spin column as described by the manufacturer's protocol. The extracted RNA was eluted in a total volume of 60 μl of elution buffer and was stored at -70°C for further use.

### Primer design

The primers used for real-time RT-PCR amplification of PRRSV were designed using sequence data from the PRRSV N protein gene. The partial sequence of the N gene of PRRSV strain ch-1a was downloaded from GenBank (accession no. AY032626) and was aligned (using Clustal W program in the MegAlign Package (DNAStar)) with the available N gene sequences of other strains of PRRSV (EF517962, EU109503, EU880437, EU880433, EF488048, EU708726) to identify the conserved regions. Primers were designed and synthesized target on conserved regions (Table 4). The possibility of primer-dimers formations and/or self-complementary formations was calculated using PrimerSelect software (DNASTAR). The theoretical primer melting temperatures (Tm) were calculated using the Oligo Calculator Programme.

**Table 4:** Oligonucleotide primers designed for PRRSV amplification by conventional RT-PCR and one step real-time RT-PCR

		Length	
		
Primer	Sequence (5'-3')	Primer (bp)	Product (bp)
Nf	CCCGGGTTGAAAAGCCTCGTGT	22	228^a^
Nr	GGCTTCTCCGGGTTTTTCTTCCTA	24	
371f	CCCGGGTTGAAAAGCCTCGTGT	22	371^b^
371r	TGTAACTTATCCTCCCTGAATCTG	24	

^a ^Length of product amplified by one step real-time RT-PCR primer pair.

^b^Length of product amplified by conventional RT-PCR primer pair.

### Dilution end-point standard curve

Serial 10-fold dilutions of the ch-1a RNA (viral RNA concentration 9.6 × 10^5 ^copy numbers per reaction mixture, which were confirmed previously, date was not shown) were performed in DEPC-treated water to 10^-7 ^with the purpose of ascertaining the detection limit. The sensitivity and reproducibility of the one step SYBR green real-time RT-PCR detection method were calculated. The lowest viral titer at which PRRSV was detectable was assigned as the detection limit. The means of the threshold cycle (Ct) for these 10-fold dilutions were used to determine minimum RNA copy numbers. Ct represents the number of cycles in which fluorescence intensity is significantly greater than background fluorescence and is directly proportional to log10 of its corresponding copy numbers value. Those samples with a Ct value below the negative control Ct value were considered as positive.

### Conventional RT-PCR

The conventional RT-PCR assay was performed in a single-step RNA extracted from field samples. A 371-bp fragment was amplified by one-step RT-PCR by using the 371f and 371r primers described in Table 1. The RT-PCR was performed in a MyCycler thermal cycler (Eppendorf). 25 μl reaction mixture contains 10 pmol of forward and reverse primers, 12.5 μl PrimeScript One-step RT-PCR reaction mix (TAKARA), and 1 μl PrimeScript One-step RT-PCR enzyme mix. The RT-PCR conditions were as follows: an initial reverse transcription for 30 min at 50°C, followed by a PCR activation for 3 min at 94°C, 30 cycles of amplification (50 s at 94°C, 50 s at 56°C, and 1 min at 72°C), and a final extension step at 72°C for 8 min. The resulting PCR products were analyzed by electrophoresis on an ethidium bromide stained 1.5% agarose gel.

### One step SYBR green real-time RT-PCR

One step SYBR green real-time RT-PCR amplification was carried out with Mx3005P Real-Time PCR System (Agilent Stratagene, USA). After optimization, PRRSV was diluted ten-fold serially to 10^-7 ^and were assayed in a 25 μl reaction mixture containing 2 μl of diluted RNA; 0.4 μM of Nf primer and 0.4 μM of Nr primer; 12.5 μl of 2× One Step SYBR RT-PCR Buffer; 1 μl of PrimeScript™ 1 Step Enzyme Mix; 7.5 μl of RNase Free dH_2_O. RT conditions were as follows: 30 min at 50°C and 2 min at 95°C, followed by 40 cycles of PCR for 30 s at 94°C for denaturation, 20 s at 56°C for annealing and 20 s at 72°C for extension. Fluorescence was detected at the end of the 72°C segment in the PCR step. The results were analysed by using MxPro™ QPCR Software.

### Melting curve analysis

After 40 amplification cycles, a melting analysis was carried out to verify the correct product by its specific melting temperature (Tm). The thermal profile for melting curve analysis consisted of a denaturation for 1 min at 95°C, lowered to 55°C for 30 s and then increased to 95°C with continuous fluorescence readings.

## Competing interests

The authors declare that they have no competing interests.

## Authors' contributions

HT carried out the molecular genetic studies, participated in the sequence alignment and drafted the manuscript. JYW participated in the sequence alignment. YC, YJS and XTL participated in its design and coordination. All authors read and approved the final manuscript.
